# *Clostridium beijerinckii* strain degeneration is driven by the loss of Spo0A activity

**DOI:** 10.3389/fmicb.2022.1075609

**Published:** 2023-01-10

**Authors:** Jonathan R. Humphreys, Bisrat J. Debebe, Stephen P. Diggle, Klaus Winzer

**Affiliations:** ^1^BBSRC/EPSRC Synthetic Biology Research Centre (SBRC), School of Life Sciences, University Park, The University of Nottingham, Nottingham, United Kingdom; ^2^DeepSeq, Centre for Genetics and Genomics, The University of Nottingham, Nottingham, United Kingdom; ^3^Center for Microbial Dynamics and Infection, School of Biological Sciences, Georgia Institute of Technology, Atlanta, GA, United States

**Keywords:** strain degeneration, solventogenic clostridia, ultra-deep sequencing, comparative genomics, biofuels

## Abstract

Solventogenic clostridia represent a diverse group of anaerobic, spore-forming bacteria capable of producing acetone, butanol and ethanol through their unique biphasic metabolism. An intrinsic problem with these organisms however is their tendency to degenerate when repeatedly subcultured or when grown continuously. This phenomenon sees cells lose their ability to produce solvents and spores, posing a significant problem for industrial applications. To investigate the mechanistic and evolutionary basis of degeneration we combined comparative genomics, ultra-deep sequencing, and concepts of sociomicrobiology using *Clostridium beijerinckii* NCIMB 8052 as our model organism. These approaches revealed *spo0A*, the master regulator gene involved in spore and solvent formation, to be key to the degeneration process in this strain. Comparative genomics of 71 degenerate variants revealed four distinct hotspot regions that contained considerably more mutations than the rest of the genome. These included *spo0A* as well as genes suspected to regulate its expression and activity. Ultra-deep sequencing of populations during the subculturing process showed transient increases in mutations we believe linked to the *spo0A* network, however, these were ultimately dominated by mutations in the master regulator itself. Through frequency-dependent fitness assays, we found that *spo0A* mutants gained a fitness advantage, relative to the wild type, presumably allowing for propagation throughout the culture. Combined, our data provides new insights into the phenomenon of clostridial strain degeneration and the *C. beijerinckii* NCIMB 8052 solvent and spore regulation network.

## Introduction

The ever increasing need to switch away from crude oil reliance makes biological production of solvents an attractive alternative, especially production of biobutanol. Butanol as a biofuel has several advantages over traditional bioethanol due to its low vapor pressure and ability to be used directly in vehicles without the need for modifications or blending (Szulczyk, 2010; Köpke et al., 2011). Solventogenic *Clostridia* represent a diverse group of bacteria known for their ability to produce the solvents acetone, butanol and ethanol from sugars, through their unique biphasic fermentative metabolism (known as ABE fermentation; Jones and Woods, 1986; Lee et al., 2008). In the first phase of their growth, they produce the organic acids acetate and butyrate as they grow exponentially. Acidogenesis provides the greatest ATP gain from sugar metabolism, however, excess production of acids leads to a drop in the pH of the medium which eventually becomes toxic to the cell. To alleviate this toxicity, these acids are partially re-assimilated and converted to the aforementioned solvents (Jones and Woods, 1986; Lee et al., 2008). The advent of solventogenesis also commits the cell to spore formation, which sees cells undergo a morphological change resulting in heat-resistant endospores for long term survival (Dürre and Hollergschwandner, 2004; Dürre, 2014).

Both solvent and endospore formation are controlled by Spo0A which plays a crucial role in the life cycle of solventogenic clostridia (Ravagnani et al., 2000). For *Clostridium acetobutylicum*, activation of Spo0A was shown to be *via* direct phosphorylation of the protein from orphan histidine kinases (Steiner et al., 2011). Furthermore, more recent studies of the histidine kinases of *C. beijerinckii* also suggest that this may be a common feature of solventogenic clostridia (Xin et al., 2020). Once activated, phosphorylated Spo0A binds to DNA sequences known as ‘0A’ boxes, found in the 5′ region of regulated genes, causing gene transcription or repression (Wilkinson and Young, 1995; Ravagnani et al., 2000; Steiner et al., 2011). In this case, transcription of genes responsible for acidogenesis are repressed whereas for genes involved in solvent and spore formation, transcription is enhanced (Wilkinson and Young, 1995; Ravagnani et al., 2000; Steiner et al., 2011).

Although solventogenic clostridia have great potential for industrial production of solvents, an intrinsic problem with these organisms is their tendency to degenerate (Kashket and Cao, 1995). This phenomenon sees these organisms lose their ability to produce solvents and form endospores and typically occurs when the bacteria are repeatedly transferred, as seen in batch fermentations, but also when grown continuously (Kutzenok and Aschner, 1952; Finn and Nowrey, 1959; Stephens et al., 1985; Hartmanis et al., 1986; Jones and Woods, 1986; Reysenbach et al., 1986; Woolley and Morris, 1990; Kashket and Cao, 1995; Chen and Blaschek, 1999; Lee et al., 2008). Although there is no reported definitive cause of degeneration, several factors have been suggested such as excessive acidification of the culture medium without sufficient time to induce the solvent genes (Kashket and Cao, 1995), growth rate of the culture (Stephens et al., 1985; Evans et al., 1998), segregational loss of strains containing a megaplasmid with the necessary solvent genes (Cornillot et al., 1997) and more recent studies linking a lack of CaCO_3_ needed for a variety of different cellular processes, to degeneration (Lv et al., 2016). In principle, a degenerate phenotype can be caused by loss or mutation of genes encoding enzymes or structural genes involved in solvent or spore formation. Alternatively, essential regulatory genes and signal transduction cascades may also be affected. The former appears to be a main course of degeneration in *C. acetobutylicum*, where loss of the megaplasmid pSOL1 disables acetone and butanol formation degeneration (Lee et al., 2010), whereas reports of spontaneous *spo0A* mutations in other solventogenic species points towards a role of the latter (Foulquier et al., 2019). Understanding degeneration is not only important for improving the economic viability of solventogenic clostridia for biofuel production but also improving our fundamental understanding of these organisms.

With the ever-increasing use of bacteria for industrial bioprocesses, it is important to understand both how and why undesirable mutants arise within a population. Fermentation homogeneity and stability is important for both the longevity of the industrial bioprocess and maximal products titers (Rugbjerg and Olsson, 2020). Non-producer cells are routinely identified in industrial fermentations and represent an important challenge to biological fermentation scale up (Zelder and Hauer, 2000; Cavaliere et al., 2017; Rugbjerg et al., 2018; Rugbjerg and Sommer, 2019). Although fermentations may begin homogeneous, non- or low-producing sub-populations arise that avoid the costly metabolic burden required for production of desired products (Rugbjerg and Olsson, 2020). Existence of these sub-populations may be due to spontaneous mutations, social cheating or product toxicity (West et al., 2007; Couce et al., 2013; Cavaliere et al., 2017; Rugbjerg et al., 2018; Couce and Tenaillon, 2019). Strain engineering to improve stability, adjustment of fermentation conditions and host selection (Adrio and Demain, 2006; Lee and Kim, 2015; Rugbjerg and Olsson, 2020) may help to overcome these barriers, however, understanding why these sub-populations arise from a more fundamental prospective may also help with prevention.

Degeneration of solventogenic *Clostridia* is another example of undesirable sub-populations arising during industrial fermentations. Since there is no definitive universal explanation as to how and why solventogenic strains degenerate, we sought to understand this process more thoroughly using a previously utilized industrial strain, *Clostridium beijerinckii* NCIMB 8052 (Stephens et al., 1985; Adler and Crow, 1987; Woolley and Morris, 1990; Kashket and Cao, 1995; Shi and Blaschek, 2008). With limited published use of modern genomic techniques to study degeneration, we employed a combination of comparative genomics, mutation accumulation (MA) with whole genome sequencing (WGS; MA-WGS; Lynch et al., 2008; Dillon et al., 2015) and ultra-deep sequencing to monitor genetic changes throughout the degeneration process. Concepts of sociomicrobiology, such as fitness assays (West et al., 2006; Ross-Gillespie et al., 2007; West et al., 2007; Darch et al., 2012), were also employed to provide an explanation as to why mutated variants accumulated within degenerated populations.

## Results

### Subculturing causes changes in culture solvent productivity

**Figure 1:**
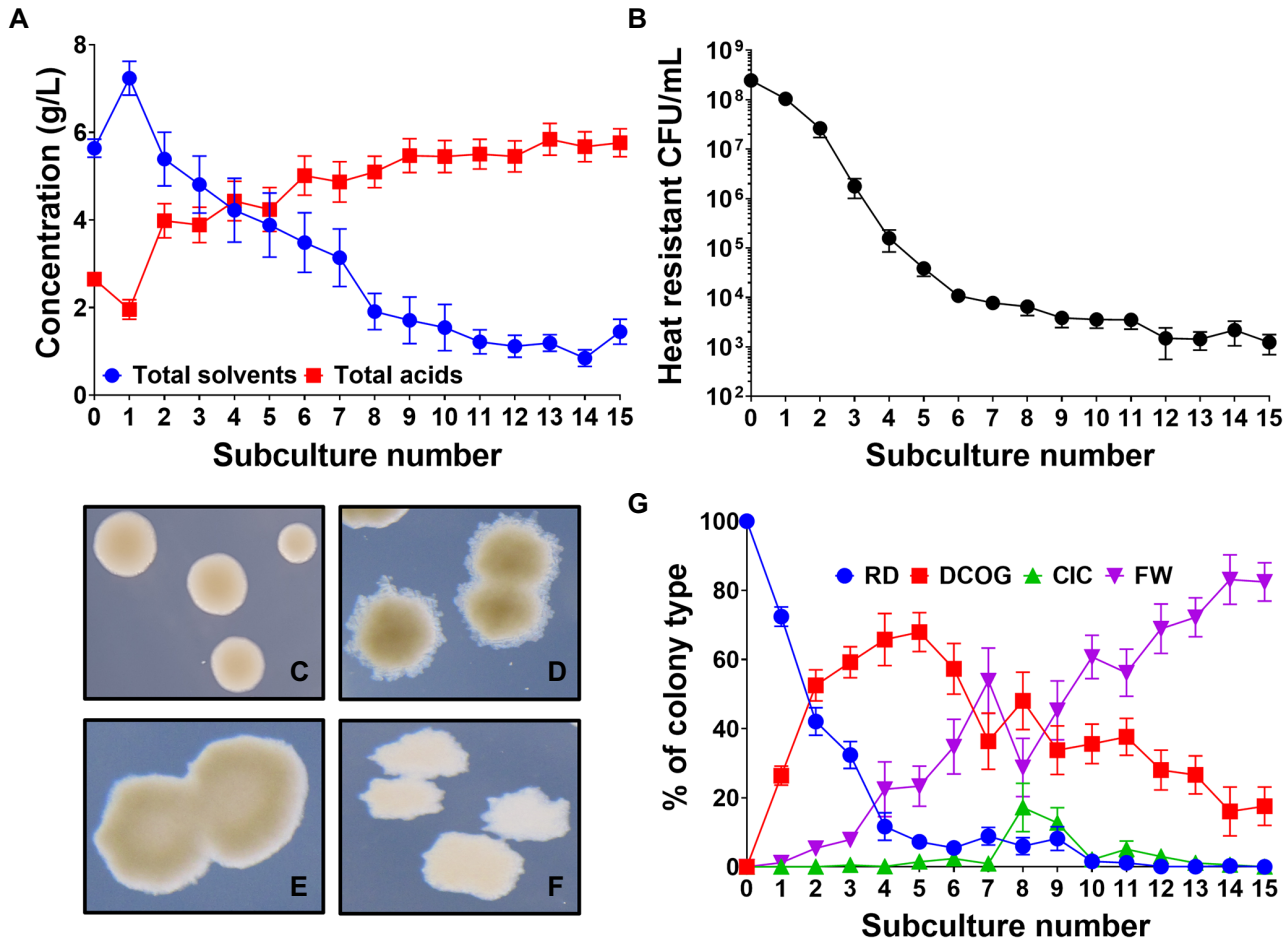
*Clostridium beijerinckii* culture dynamics throughout the degeneration process. **(A)** Concentration of fermentation products (acetone, ethanol, butanol, acetate, and butyrate) produced by the whole culture at each successive subculture. **(B)** Number of heat resistant spores produced by the whole culture at each successive subculture. **(C–F)** Four different colony types observed: **(C)** “round and dark” (RD), **(D)** “dark centre and outgrowths” (DCOG), **(E)** “caved in centres” (CIC), and **(F)** “flat and white” (FW). **(G)** Percentage of each colony type found during subculturing. Error bars represent standard error of the mean (± SEM). *n* = 16, for **A,B,G**.

As subculturing has been employed to promote degeneration of solventogenic clostridia in the past (Kutzenok and Aschner, 1952; Hartmanis et al., 1986; Hayashida and Yoshino, 1989; Chen and Blaschek, 1999), we established our own subculturing protocol using wild-type (WT) *C. beijerinckii* NCIMB 8052, to promote degeneration of this strain and reproduce previously reported findings (see Supplementary Figure 1 for schematic). This protocol involved repeatedly subculturing WT *C. beijerinckii* at 24-h intervals, in a 1 in 10 dilution. Each subculture series was initially inoculated with a colony derived from a single heat-shock germinated spore to generate a genetically homogeneous population and remove any cells that had not formed functional spores (and therefore had already degenerated). We found that at each successive subculture, a consecutive decrease in the concentration of solvents (acetone, butanol and ethanol) produced by the culture was observed. This was mirrored by a steady increase in the production of organic acids (acetate and butyrate; Figure 1A). We found that after 15 subcultures, the total concentration of solvents had decreased 80% from a maximum of 7.23 ± 0.3 g/L at subculture 1 to 1.44 ± 0.26 g/L at subculture 15. Conversely in the same time frame, organic acids increased by 195% from 1.95 ± 0.22 g/L to 5.76 ± 0.32 g/L. Alongside a successive decrease in solvents, the number of viable heat resistant spores (i.e., functionally germinating spores) produced by the culture also decreased as the subculturing progressed, correlating with the decrease in solvents (Figure 1B). It should be noted that the lower solvent concentrations found at subculture 0 reflect the fact that each replicate tube is inoculated with a single colony compared to a larger inoculum from a full-grown culture which will affect the final solvents produced.

### Subculturing showed dynamic changes in colony morphology

Much of the early studies into degeneration commented on aberrant colony morphologies emerging that would appear misshapen when compared to the WT morphology (Adler and Crow, 1987; Kashket and Cao, 1995). In the aforementioned subculturing experiment (Figure 1), we observed the colony morphologies of subcultured *C. beijerinckii* to assess whether colonies had undergone any morphological changes. We found four distinct colony morphologies emerged in the population which we named the round and dark (RD; Figure 1C), which is the WT morphology, the dark center with outgrowths (DCOG; Figure 1D), the caved in center (CIC; Figure 1E) and the flat and white (FW; Figure 1F). With four colony types established, we monitored their appearance throughout the subculturing process, which showed dynamic changes in their percentage of the whole culture (Figure 1G). In the early subcultures, the amount of observed RD within the population rapidly decreased with the advent of the DCOG at subculture 1. This type began to take over the population occupying around 70% of the observed CFU/ml by subculture 5. Although the DCOG quickly enriched within the culture, on average this increase was not maintained past the subculture 5 point as we observed, though less rapid, an incremental increase in the numbers of FW also from subculture 1. This type eventually outcompeted all others and by the end of the subculturing experiment (15 subcultures), occupied over 80% of the observed CFU/ml. We did on occasion for some replicates observe a sudden increase in the CIC at subculture 8, which appeared to promote DCOG competitiveness, however CIC CFU/ml was not maintained, and numbers of CIC decreased after this point. At the end of the subculturing, the final population consisted mostly of FW, with some DCOG still present. Neither the CIC nor the RD remained at this point, suggesting that the WT colony morphology was below the limit of detection.

### Phenotypic assessment of the identified colony types

Once we had established that four colony morphotypes existed through subculturing, we isolated a total of 17 RD, 17 DCOG, 18 CIC, and 19 FW in two separate rounds of isolation. Both rounds of isolation began with a single, WT *C. beijerinckii* colony that was subcultured 5 times. Single colonies were generated from germinated spores which were used to not only remove any variants unable to produce viable spores but also to provide the starting genomic profile which any subsequent mutations could be distinguished from. These single germinated spore colonies were then serially transferred five times and cultures were plated on CBMS agar to reveal the different colony morphotypes for isolation. We then phenotypically characterized each of the 71 variants based on their fermentation profiles (after 2 days of growth) and level of sporulation (after 5 days of growth) to ascertain the level, if any, of degeneration each variant had undergone (Supplementary Figures 2, 3). We found that generally, although there were some outliers, the RD, DCOG, and CIC fermentation profiles were similar as variants of these types produced solvents to a comparable degree to the WT (Supplementary Figure 2). The FW morphotype however showed the most drastic solvent phenotypes as only four of this type were able to produce comparable solvents to the wild type and the rest produced little to none (Supplementary Figure 2). We found however, far more homogeneity between morphotypes regarding their sporulation profiles as all variants, besides one RD, produced significantly less spores than the wild type even if their solvent profiles appeared normal (Supplementary Figures 2, 3). Again, the FW showed the lowest production of viable spores with only four variants capable of producing, albeit to a lower degree, viable spores (Supplementary Figure 3D). The profiles of each of the variants appeared to show that although solvent production and sporulation are often linked, normal levels of solvents can still be achieved for some mutants regardless if sporulation is impaired.

### Whole genome sequencing revealed four distinct hot spots of mutations

After phenotypic characterization of the variants, we genotypically characterized each variant by comparing their genome sequences to the parental WT they were originally derived from. Any mutations found within the parental WT were removed from mutations found in the variants due to their existence before subculturing. Genetic analysis revealed a total of 211 SNPs (single nucleotide polymorphism), 12 deletions and 2 insertions after five serial transfers (equating to approximately 16–17 generations starting from the subculture 0 population) for the 71 variants sequenced. The average number of mutations per degenerate was therefore 3.16 after the five serial transfers. The transversions G- > T and C- > A were over-represented as they made up 87% of the observed SNPs. A full table of the mutations found for each variant can be found in Supplementary Table 1 and provides a list of candidate genes for not only degeneration, but key regulatory elements in *C. beijerinckii* NCIMB 8052.

**Figure 2:**
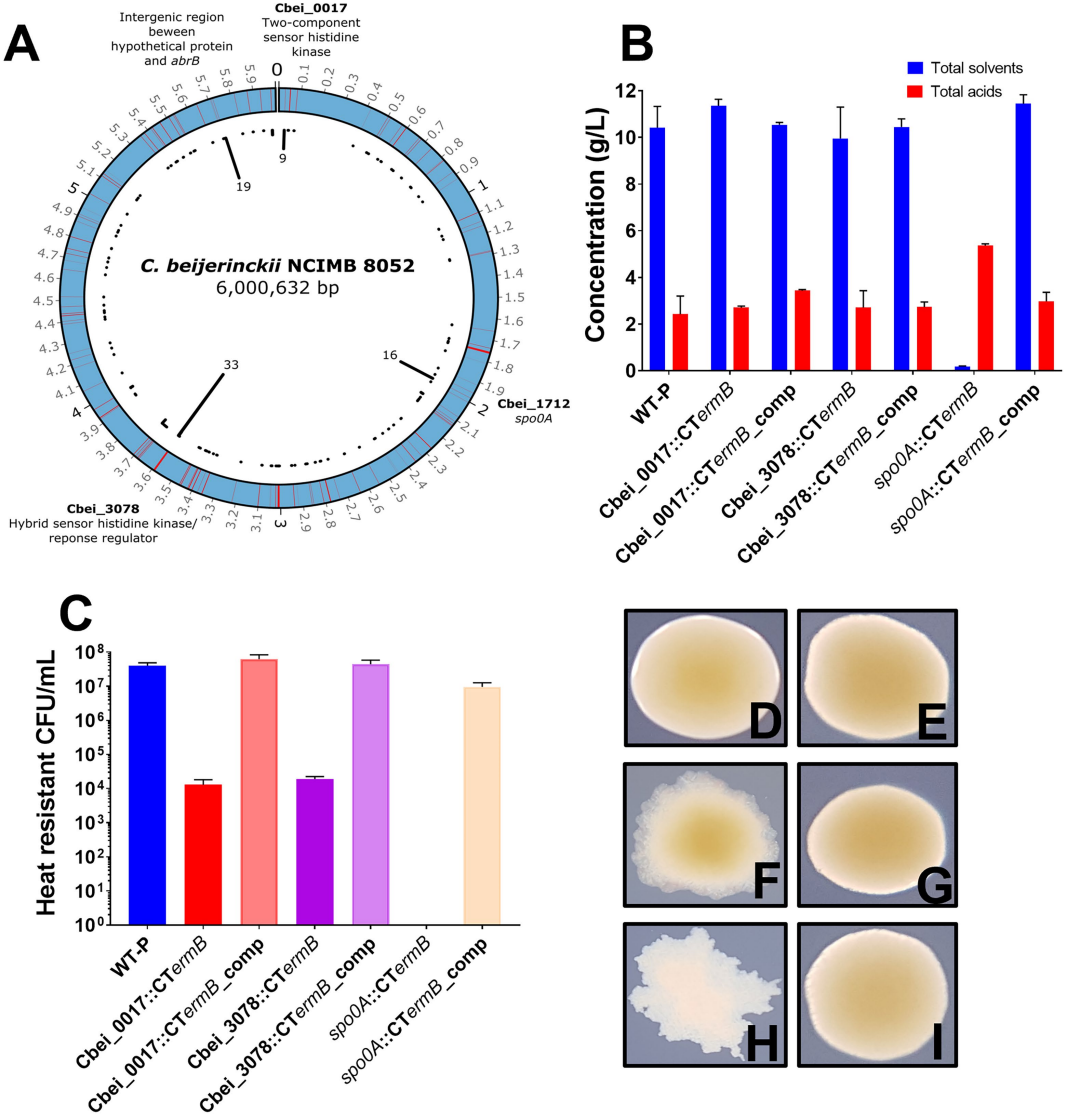
Genomic distribution of mutations in degenerate isolates and ClosTron mutant characterisation. **(A)** Image representing the distributions of mutations along the circular *Clostridium beijerinckii* NCIMB 8052 chromosome found for all degenerate isolates. Red lines indicate where a mutation was found and black lines in the centre of the image show the number of times that region was hit. Genomic regions that contained significantly more mutations than others are highlighted. **(B)** Fermentation products produced by *spo0A,* Cbei-0017 and Cbei_3078 ClosTron mutants and complemented strains. **(C)** Spores produced by ClosTron mutants and complemented strains. **(D–I)** ClosTron and complemented mutants colony morphotypes: **(D)** Cbei_0017 mutant, **(E)** Cbei_0017 complemented mutant, **(F)** Cbei_3078 mutant, **(G)** Cbei_3078 complemented mutant, **(H)** *spo0A* mutant and **(I)** *spo0A* complemented mutant. Error bars represent standard error of the mean (± SEM) of three independent experiments, each performed in technical triplicates.

Four distinct “hot spot” regions emerged that contained significantly more mutations than elsewhere in the genome. These regions included a two-component sensor histidine kinase (Cbei_0017; hotspot region 1), the master regulator gene *spo0A* (Cbei_1712; hotspot region 2), a hybrid sensor histidine kinase/response regulator (Cbei_3078; hotspot region 3) and a final region that contained a hypothetical protein-encoding gene (Cbei_4884), a neighboring *abrB* (Cbei_4885) transcriptional regulator gene, as well as the intergenic region in between (hotspot region 4). To confirm that the mutations were present in our variants and not an artifact of our genome sequence analysis, we chose five random mutants from each hotspot region to PCR amplify chromosomal DNA at each hotspot region. All PCR product bands were subjected to Sanger sequencing which confirmed that the specific mutations were all present. Figure 2A shows the distribution of the mutations throughout the circular *C. beijerinckii* chromosome. Furthermore, the position of the mutations in the hotspots relative to the position in the gene and protein can be found in Supplementary Figure 4.

There was some link between colony morphotype and genotype. Out of 9 mutations found in Cbei_0017, 7/9 of those were of the RD morphotype. For 21 unique variants that had mutations in Cbei_3078, over half (12/21) were of the DCOG morphotypes and 12 out of the 16 *spo0A* mutants were of the FW morphotype. The CIC morphotype appeared to represent a less defined link between colony morphology and genotype as variants of this type had mutations in all the hotspots (although not all at once) and so may well embody a morphotype that is less strictly associated with mutations in individual genes.

### ClosTron mutagenesis for mutation validation

We hypothesized that the two identified histidine kinases (Cbei_0017 and Cbei_3078) may be involved in Spo0A regulation in *C. beijerinckii* NCIMB 8052, as in the closely related species, *C. acetobutylicum* ATCC 824, direct phosphorylation by orphan histidine kinases has been shown to regulate Spo0A activity (Steiner et al., 2011). ClosTron (Heap et al., 2007, 2010) intron insertions were employed to functionally inactivate Cbei_0017, Cbei_3078 and as a control, *spo0A,* generating the mutants Cbei_0017::CT*ermB*, Cbei_3078::CT*ermB* and *spo0A*::CT*ermB*, respectively. Furthermore, we subsequently complemented any mutants by inserting a functional copy, including the native promoter, of the inactivated gene back on the chromosome at the *pyrE* locus (Ng et al., 2013; Ehsaan et al., 2016) to confirm their role. As expected, we found that the *spo0A* intron insertions completely removed solvent and spore formation (Figure 2B). In contrast, the Cbei_0017 and Cbei_3078 insertion mutants were still capable of producing solvents at a similar concentration to the WT but did produce reduced spore counts (Figures 2B,C). Complementation by inserting intact copies of the genes at the *pyrE* locus on the chromosome restored lost phenotypes due to the ClosTron insertions (Figures 2B,C).

As with the naturally derived mutants, we observed an interesting link between colony morphology and genotype as the ClosTron mutants showed a remarkable similarity to the previously discovered colony morphologies. Cbei_0017::CT*ermB* mutants appeared as the RD, and complementation of Cbei_0017 had no effect on morphology (Figures 2D,E). Cbei_3078::CT*ermB* mutants however appeared like the DCOG morphotype, and complementation back onto the chromosome restored WT colony appearance (Figures 2F,G). *spo0A*::CT*ermB* mutants appeared as the FW morphotype and again, complementation of the *spo0A* back onto the chromosome restored the colony morphotype back to normal (Figures 2H,I). These results further illustrated the role that these genes play for both physiological characteristics but also on colony morphology. The observed findings here support what we found for the subculture experiments as losing Cbei_0017 and Cbei_3078 activity had little effect on solventogenesis but severe effects on sporulation.

### Ultra-deep sequencing showed dynamic genotypic changes

**Figure 3:**
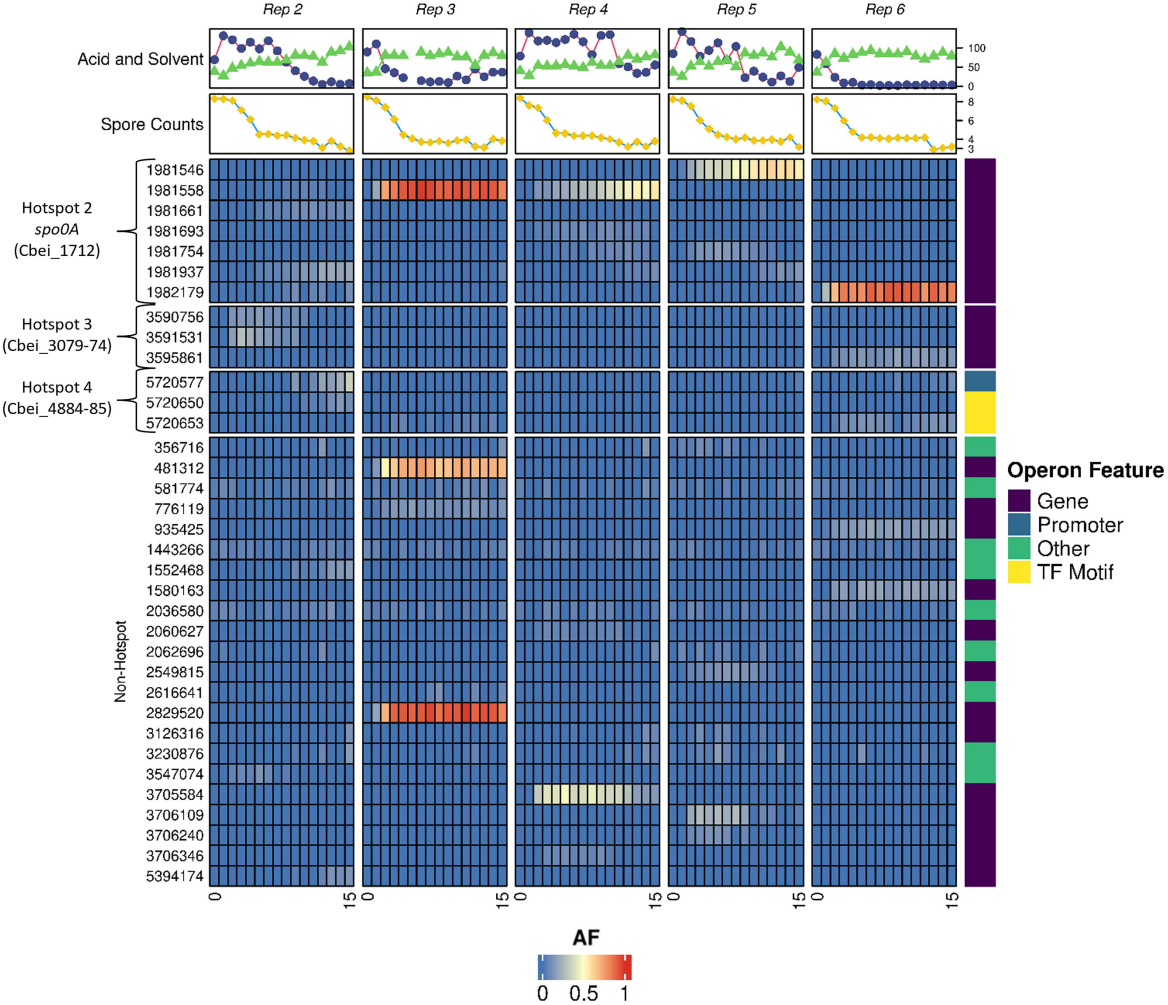
Allele frequency dynamics in degenerating *Clostridium beijerinckii* cultures. Heatmap showing the frequency of mutations emerging within the populations of 5 individual subculture series. Subculture numbers for each series are indicated at the bottom of map. AF is the allele frequency. Hotspot regions 2, 3, and 4 are shown along with non-hotspot regions where other mutations were found. Solvent (Blue circles) and acid (Green triangles) concentrations (mM) alongside spore counts (10^x^ heat resistant CFU/ml; yellow squares) are displayed for each individual subculturing point. Operon feature shows whether the mutations affects the gene, promoter region, transcription factor motif or other region. All variants called for the heatmap can found in Supplementary Table 4B. Median read depth reported by Freebayes and Lofreq was 368 and 385, respectively.

Ultra-deep sequencing was employed to track mutations within the degenerated populations to both understand the genetic dynamics of the subculturing process and to observe if and when our discovered hotspots gained or lost mutations within the whole culture. With this technology, it was possible to detect specific mutations when present within 4% or more of the population. Subculturing was again repeated in 5 independent parallel replicates and whole culture genomes, solvents, acids and spore samples were taken at each subculture point to provide an insight into both the genetic and phenotypic make-up of the culture at specific subcultures. Raw allele frequency (AF) numbers as determined for each subculture can be found in Supplementary Table 4 and were used to generate Figure 3.

Although mutations in hotspot regions 3 (Cbei_3078 operon) and 4 (the hypothetical protein and *abrB* region) were found, frequencies in these regions were less, relative to the colony sequence comparisons, as the frequency of mutations in these regions never reached above 50% (Figure 3.). This may in part be due to an overrepresentation of mutations in these regions at the colony sequencing stage when we initially selected colonies based on morphotype, rather than at random. Mutations of *spo0A* were found in significant frequencies and their appearance generally coincided with a decline in solvent formation by the cultures (Figure 3). Interestingly, no mutations in hotspot region 1 (Cbei_0017) were found, indicating that as with regions 3 and 4 it may have been overrepresented due to the morphotype selection bias during the single variant selection. It is clear however that the *spo0A* mutations dominated the subculturing process, and that the advent of these mutations appears to outcompete other mutations within the culture genomic pool. This is evident by the dynamic changes seen for some of the hotspot mutations that are lost by the end of the subculturing process, such as those seen for region 3 in replicate 2 (a similar trend as seen when observing the colony morphotypes). Interestingly, replicate 2 showed multiple *spo0A* mutations which we suspect all contributed to a loss in solvent formation, compared to a dominant single mutation seen for the other replicates. Elevated frequencies of mutations found in regions outside of the determined hotspots appear as carrier mutations that we suspect are found within the same mutants of *spo0A*, which are only enriched to high numbers due to increases in *spo0A* mutants.

Most of the mutations found were within genes, however, several mutations appeared to affect promoter or transcription factor binding regions (Figure 3). One notable promoter region mutation was found upstream of the *abrB* gene (Cbei_4885) at position 5,720,650 and 5,720,653. Mutations at position 5,720,653 have been reported previously in a variant of *C. beijerinckii* (BA105; Seo et al., 2017). This strain showed degeneration phenotypes as it made significantly more acids and less solvents compared to the WT. Sequence analysis of this region by Seo et al. (2017) revealed a putative 0A box with the sequence TGTCGAA (Ravagnani et al., 2000). The emergence of mutants in these regions suggests that not only mutations within genes, but mutations in promoter/0A box regions contribute to the emergence of degenerates.

To confirm that the mutations found from the ultra-deep sequencing were indeed dominating within the evolved populations, we took 10 random colonies from the final subculture for each of the 5 replicate cultures and whole genome sequenced these colonies to determine the mutations present at the end of the experiment. A full list of all the mutations for these colonies can be found in Supplementary Table 2. Indeed, we found that many of the individual colonies isolated from the terminal subculture of each replicate carried the same mutations as identified by ultra-deep sequencing, thus confirming the validity of our approach.

### *spo0A* mutants have a greater fitness than WT and other strain variants

**Figure 4:**
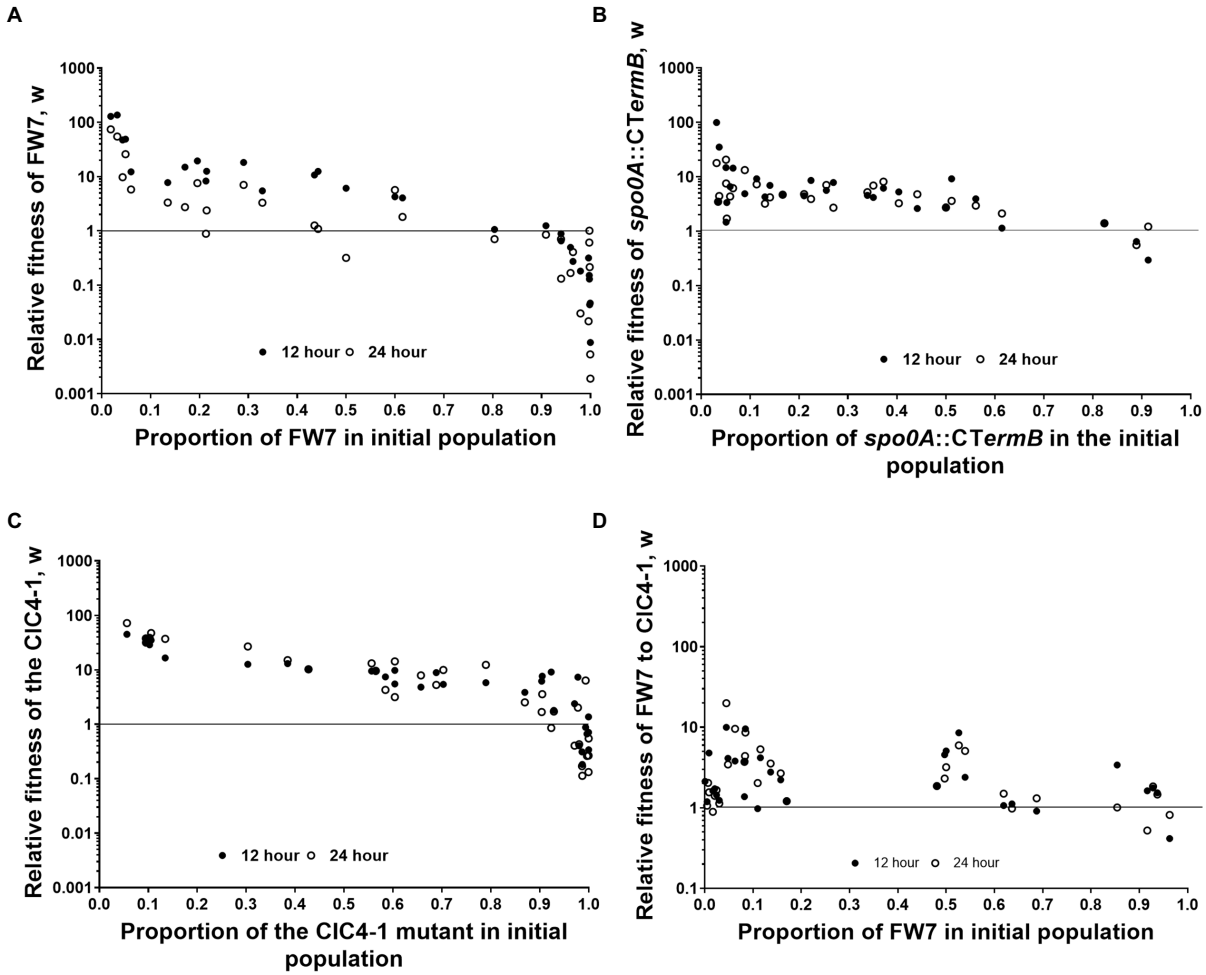
Fitness assays between WT and derived mutants. Frequency-dependent assay of **(A)** FW7 vs. WT, **(B)** spo0A::CTermB vs. WT, **(C)** CIC4-1 vs. WT, and **(D)** FW7 vs. CIC4-1. Data points represent individual mixed cultures. Solid circles are 12 h values and empty circles are 24 h values. *n* = 30.

As we found that Spo0A, and the network that we suspect to regulate this protein, seem to play a key role in degeneration, we wanted to test whether loss of Spo0A activity gave a measurable fitness advantage. To do this we initially took a naturally derived *spo0A* mutant (named FW7) which contained two SNPs, one being a silent mutation within Cbei_2,920 and another within the specific DNA binding motif encoding part of *spo0A*^7^, rendering the protein nearly inactive as this strain produced less than 1 mM solvents and no spores (Supplementary Figures 2, 3). We subsequently mixed FW7 and the WT in various starting ratios, observing the CFU/ml of each at 0, 12, and 24 h after mixing. The relative fitness of FW7 to the WT was calculated based on Diggle et al. (2007; see section Materials and methods) with values above 1 indicating that the mutant is fitter than the WT. FW7 showed a high relative fitness, compared to the WT, when it occupied between 1% and 60% of the initial culture (Figure 4A). Relative fitness values were highest when the proportion of FW7 in the initial population was the least, and as the proportion of FW7 was increased, relative fitness values began to decrease for both the 12 and 24 h values. FW7 became as fit, or less fit, than the WT (relative fitness values of 1 or less) when the mutant occupied around 80% or more of the initial population (Figure 4A). These results are suggestive of negative frequency-dependent fitness (and perhaps some form of social cheating) as mutants are expected to do better when they are rare as there are more cooperating cells for them to exploit (Ross-Gillespie et al., 2007).

To further confirm that loss of *spo0A* can provide a measurable fitness increase, we repeated the same fitness experiment but used a ClosTron inactivated *spo0A* mutant (*spo0A*::*CTermB*) in place of FW7 (Figure 4B). Again, we found a very similar trend as low amounts of *spo0A*::*CTermB* in the initial population gave very high relative fitness values. These decreased as the initial population of the mutant was increased. Furthermore, *spo0A*::*CTermB* fitness become the same or less fit to the wild type when the mutant occupied above 80% of the population, the same as FW7. This further confirmed the considerable fitness benefit conferred by losing Spo0A functionality.

To test whether partial loss of *spo0A* activity would give similar results, we chose CIC4-1 as this variant had a mutation in the suspected phosphoacceptor domain of Spo0A (Brown et al., 1994), which allowed the mutant to produce some solvents but was unable to produce spores (Supplementary Figures 2, 3). This mutant had also gained SNPs within a non-coding region and an annotated galactose transporter (Cbei_3422) which we assumed did not play a significant role in degenerate phenotypes. Again, a similar frequency-dependent trend was seen for this mutant, mirroring what we observed for FW7 and *spo0A::CTermB* (Figure 4C). Interestingly however, CIC4-1 was able to sustain higher relative fitness values for longer than the other two mutants when the initial proportion was increased, showing values above 1 even when reaching 90% of the population.

We then tested the fitness between FW7 and CIC4-1 to observe whether full or partial loss would give the highest fitness values. We found that the relative fitness of FW7 was generally higher than that of CIC4-1 although there was not as clear evidence of frequency-dependent fitness as seen with the WT mixed cultures (Figure 4D). This still suggests however that complete loss of Spo0A activity does give the greatest fitness advantage, however, this advantage is less pronounced when grown against a strain carrying partially active Spo0A.

### Adjusting the subculture regime or applying a heat shock bottleneck improves culture stability

**Figure 5:**
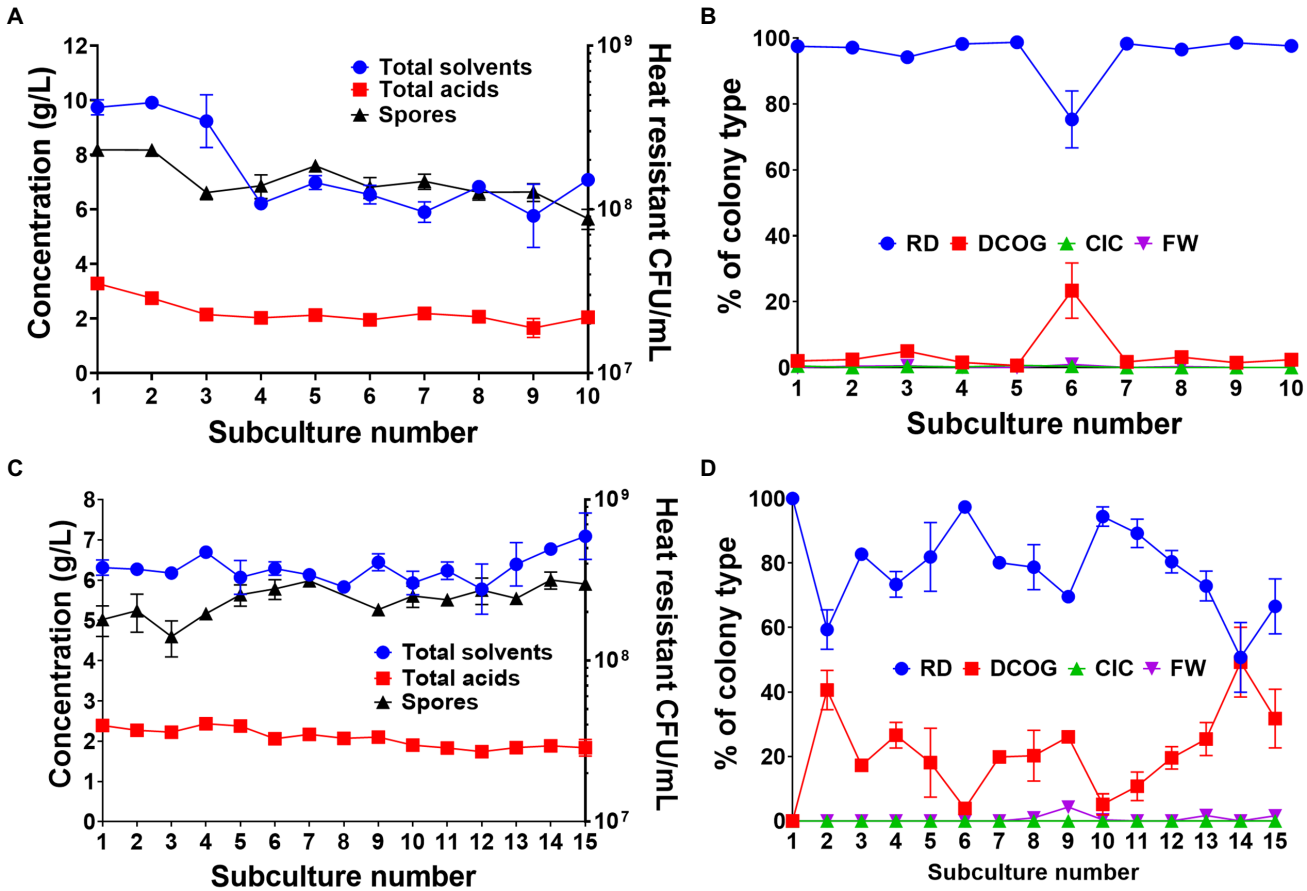
Effects of 72 h transfer time **(A,B)** and applying a heat shock bottleneck before subculturing **(C,D)** on culture productivity, spore formation and colony morphotype dynamics. **(A)** Concentration of fermentation products (combined solvents: acetone, ethanol, butanol, combined acids: acetate and butyrate) and number of heat resistant spores produced by the whole culture at each successive 72 h subculture. **(B)** Percentage of each colony type found during the 72 h subculturing. **(C)** Concentration of fermentation products (combined solvents: acetone, ethanol, butanol, combined acids: acetate and butyrate) and number of heat resistant spores produced by the whole culture at when a heat shock is applied before inoculation of each subculture. **(D)** Percentage of each colony type found when a heat shock is applied at each subculture. Values are the means (± SEM) of the biological replicates. *n = 5* for the 72 h transfer and *n* = 3 for the heat shock transfers.

During the fitness assay tests, we observed a rapid decline in the CFU/ml of FW7 after more than 24 h of growth (Supplementary Figure 5). We hypothesized that although the mutant had gained considerable fitness compared the WT, this fitness only provided an advantage within the early stages of growth and could not be maintained as cell entered the stationary phase. We attempted to improve culture viability by repeating our standard subculturing experiment but changing the transfer time from 24 to 72 h. As before, we started with single, germinated WT spores which we inoculated into 10 ml of medium and subcultured every 72 h in a 1/10 dilution for five independent biological replicates. Using the 24 h transfers as a comparative control, solvent production was maintained throughout the whole subculturing process (Figure 5A), unlike what we observed previously (Figure 1). We did observe a drop in solvent production following subculture 3, however, after this point solvents stabilized at around 6 g/L. Furthermore, the number of spores produced by the cultures remained relatively stable throughout the whole experiment (Figure 5A). Another interesting observation was the maintenance of the RD type which remained consistently as the major colony type throughout the subculturing (Figure 5B). A small increase in the DCOG was seen at subculture 6, but this was not maintained. Adjusting the transfer time appeared to have a clear effect on the degeneration rate of the culture.

We also carried out a heat shock bottlenecking experiment to observe whether applying a bottleneck, one in which only spore-forming, un-degenerated cells could survive, to serve as a control to maintain culture stability. Subculturing was repeated at 24 h intervals for three independent replicates, however we heat shocked 1 ml of the transfer culture at 80°C for 10 min, thus inactivating all vegetative cells and inducing germination of any viable spores present. As to be expected, we found that culture stability was maintained throughout the 15 subcultures as solvent production was maintained at around 6 g/L (Figure 5C). Spore numbers were also maintained and did not drop below 10^8^ heat resistant CFU/ml throughout the experiment (Figure 5C). When we observed colony morphologies, the RD type was again the predominant type, however, the DCOG type existed in greater numbers than when the transfer time was adjusted (Figure 5D). This in part may be due to the DCOG type still capable of forming viable spores. There were little to none of the FW or CIC observed during this bottlenecking experiment.

In addition to phenotypic observations, we sequenced 10 colonies from three independent cultures found at the end of the heat shock experiment to observe whether this regime prevented the emergence of degenerate genotypes. Indeed, only one (replicate 2, colony 4) had gained a mutation linked to degeneration as this variant had mutation in *spo0A* (Supplementary Table 3). It is unclear whether this mutation was gained pre- or post-germination, however, the lack of significant degeneration genotypes exemplifies the effectiveness of this regime in preventing degeneration from occurring.

## Discussion

The phenomenon of strain degeneration sees solventogenic *Clostridium* spp. lose the ability to produce solvents and form endospores, two key processes in their growth cycle. The degeneration phenomenon is one of the key issues that needs to be solved to improve commercial use for biofuel and chemical production as well as other biotechnological applications. Although several mechanisms of degeneration have been proposed, there is no established definitive cause as to why strains degenerate., although a ‘global regulator’ has been suggested as one of the causes of strain degeneration (Kashket and Cao, 1995). Here, we confirm that the accumulation of degenerate mutants within *C. beijerinckii* cultures is linked to the loss of Spo0A activity, either through direct mutation of the encoding gene or mutations in loci we believe to be involved in Spo0A regulation. Through ultra-deep sequencing we show that *spo0A* mutations enrich and outcompete other mutations in the population, due to *spo0A* mutants showing a greater fitness to the WT. Furthermore, as one might expect, we were able to maintain the capacity to form spores and solvents through adjusted transfer times and by applying bottlenecks.

For solventogenic clostridia, Spo0A serves to regulate the crucial switch to solventogenesis and endospore formation (Ravagnani et al., 2000; Dürre and Hollergschwandner, 2004; Al-Hinai et al., 2015; Diallo et al., 2021). In one of the most characterized species, *C. acetobutylicum* ATCC 824, Spo0A has been shown to be activated through histidine kinase-mediated phosphorylation but also regulated by AbrB-type transcriptional regulators (Scotcher et al., 2005; Steiner et al., 2011; Xue et al., 2016; Seo et al., 2017). Our genomic comparisons of 71 *C. beijerinckii* potential degenerate variants showed four distinct hot spots regions containing more mutations than the rest of the genome. These included two histidine kinases (Cbei_0017 and Cbei_3078), which we suspect to be involved in Spo0A activation. Interestingly, none of the investigated histidine kinases by Xin et al. (2020) appeared in our isolates, even though they showed that by deleting Cbei_2073 and Cbei_4484, butanol production by their strain was increased, suggesting a role with *spo0A*. Genome sequencing of *C. beijerinckii* BA101 (a hyper butanol producer derived from *C. beijerinckii* NCIMB 8052) however found a SNP in Cbei_3078 causing a stop codon within the gene (Seo et al., 2021). This strain has a reduced capacity to from spores which may be explained by the mutation in Cbei_3078. Little is known about Cbei_0017, however, it has been suggested in a recent review of sporulation in solventogenic clostridia, that it may play a role in *spo0A* regulation (Diallo et al., 2021), although its precise role is currently unknown. Our ClosTron mutagenesis (and chromosomal complementation for phenotype restoration) confirmed the involvement of both loci with the *spo0A* network due to the reduced capacity of the generated mutants to form spores (Figure 2C). Interestingly, given the more than 1,000-fold reduction in spore formation, solvent production by both mutants appeared to be unchanged in comparison to the WT. We speculate that this may in part be due to differences in the Spo0A ~ P levels needed for transcription of the solvent genes to occur, as compared to those required for the initiation of sporulation. In *Bacillus subtilis*, high levels of Spo0A ~ P are required to induce sporulation and such genes also showed a low binding affinity for Spo0A (Fujita et al., 2005; Fujita and Losick, 2005). If this also applies to the clostridial Spo0A regulatory network, minor disruptions in the ability of Spo0A to be phosphorylated, such as inactivation of a single histidine kinase that phosphorylates Spo0A, may have a more profound effect on genes that require significantly more Spo0A~P than other genes. For *C. acetobutylicum*, multiple histidine kinases were shown to be involved in Spo0A phosphorylation, and so both Cbei_0017 and Cbei_3078 may need to work in conjunction to achieve full activation of Spo0A in *C. beijerinckii*. Alternatively, outside of a direct role with Spo0A phosphorylation, these two histidine kinases may phosphorylate other proteins in the sporulation cascade, rather than direct phosphorylation of Spo0A itself, leading to reduced but not abolished sporulation phenotypes when mutated. It should also be noted that asporogenous, but solvent producing cultures, have been described previously (Gottschal and Morris, 1982), which may have arisen due to mutations in genes involved in only the sporulation cascade and not the formation of solvents. Other intermediary variants have also been found that produce reduced solvents and spores but neither has been completely lost (Woolley and Morris, 1990). Further investigation is required of these histidine kinases and their combined inactivation may shed more light into their role in *C. beijerinckii* NCIMB 8052.

The hotpot region containing Cbei_4884 (a hypothetical protein) and Cbei_4885 (an *abrB* transcriptional regulator) also appears to play a role in strain degeneration. Many of the mutations we found were in the intergenic region between the genes, with several appearing to affect a putative 0A box upstream of the *abrB* gene. Interestingly, Seo et al. (2017) reported the same mutation in the 0A box upstream of *abrB* (at position 5,720,653) which we observed in both our single colony and ultra-deep sequence analysis. We suspect that mutants of this 0A box may have a reduced capacity for Spo0A to bind thus altering the expression patterns of *abrB.* In *C. acetobutylicum,* some *abrB* regulators initially help repress *spo0A* expression (Xue et al., 2016) until elevated Spo0A begins to repress *abrB* expression in a feedback loop. Without sufficient binding of Spo0A to the disrupted 0A box, continual repression of *spo0A* by AbrB will occur, potentially causing degeneration phenotypes as there is a limited switch to solvents and spore formation. This exemplifies the tight regulation in the switch, as single point mutations in the binding 0A box appear to abolish, or at least significantly reduce, the ability of Spo0A to bind and thus increase or decrease gene expression of genes controlled by Spo0A.

The role of Cbei_4884 remains unclear; however, with several variants containing mutations of this gene, alongside its proximity to Cbei_4885, there is an implication that this gene has some involvement in the regulation of sporulation and solvent formation and thus results in degeneration phenotypes when mutated. Interestingly, many other clostridial species which have Cbei_4885 homologs, also have homologs of Cbei_4884 in the same orientation suggesting a conserved role in *Clostridium* spp. Furthermore, several other potential regulatory genes appeared in our analysis including the annotated TetR regulator Cbei_3077 and Cbei_3169/3170 which appear to be linked to an *agr* quorum sensing system. Agr quorum sensing systems have been shown to affect spore formation in solventogenic species (Steiner et al., 2012). These genes would require further investigation to determine their role but do appear to have some involvement as greater than 1 variant had mutations in these genes (Supplementary Table 1).

Ultra-deep sequencing allowed us to track the emergence and disappearance of mutations during the subculturing process. Although we observed transient increases in many mutations throughout the genome, mutations in *spo0A* ultimately dominated the culture (Figure 3). The occurrence of these *spo0A* mutations also coincided with a decrease in solvent production by the culture, presumably through the lack of a functional Spo0A. Interestingly, a *spo0A* deleted strain of *C. beijerinckii* was reported to still produce solvents (3 vs. 9 g/L by the wild type; Seo et al., 2017), which is unusual as from both our studies, and others using *C. beijerinckii* NCIMB 8052, insertional and single point mutation can render mutants unable to produce solvents (Ravagnani et al., 2000). Furthermore, random single point mutations in the *spo0A* of *C. acetobutylicum*, which were reproduced in *C. saccharobutylicum*, abolished solvent formation (Foulquier et al., 2019). For most ultra-deep sequencing replicates, a single mutation in *spo0A* was found to be dominant within the population. In the case of replicate 2 however, we observed multiple mutations in the gene, suggesting that it is possible for several distinct *spo0A* mutants to coexist in the population. This was confirmed using frequency-dependent fitness assays (Diggle et al., 2007). By using a naturally evolved *spo0A* mutant (FW7) that produced little to no solvents or spores (Supplementary Figures 2, 3), we found that losing Spo0A activity gave the mutant a considerable increase in short-term fitness compared to the wild type, especially when the proportion of the mutant in the initial population was low. As we increased this proportion, we found that fitness values began to decrease which is indicative of negative frequency-dependent fitness. This was also confirmed with the ClosTron knockout of Spo0A (Figure 4B) and variant CIC4-1 as both showed a measurable fitness increase to the WT. This may explain why in replicate 2 multiple mutants existed in the population as varying levels of Spo0A activity loss still provide a fitness advantage. It would have been interesting to continue the subculturing for that replicate to observe whether a single, dominant *spo0A* mutant would eventually have taken over the population.

Frequency-dependent fitness is often attributed to social cheating, in which a mutant is capable of exploiting public or common goods in a population without contributing to their generation (West et al., 2006). It is not entirely clear what such goods would be in our scenario, although WT cells in contrast to Spo0A mutants will carry out the removal of potentially toxic acid concentrations, thereby raising the pH of the culture. Nevertheless, this only happens towards the end of growth and is therefore unlikely to fully explain the considerable increases seen for the mutant populations. We speculate that the outcompeting by Spo0A mutants may in fact be due to several alternate reasons. Firstly, without a functional Spo0A, there would be no activation of the solvent or sporulation genes, no repression of genes involved in acidogenesis and none of the typical morphological changes cells usually undergo during the switch to solvent production. This lack of morphological change we speculate may allow cells to continue to grow, produce acids and gain the maximal amount of ATP from sugar (Jones and Woods, 1986) without transitioning to non-growing sporulating cells. Secondly, continual acid production by the mutants may promote the triggering of sporulation in the WT, as acids and lower pH have been thought to contribute to the initiation of sporulation (Diallo et al., 2021). Given that *C. beijerinckii* is an efficient sporulator, non-sporulating mutants would thus be able to take over the population at this stage in the growth cycle as a major part of the WT population would enter the irreversible process of endospore formation, thereby drastically reducing the number of viable vegetative cells. Evidence of this can be seen in Supplementary Figure 5, which shows a maximum WT count of approximately 10^8^ CFU/ml, which is reduced to about 10^7^ CFU/ml following the onset of sporulation, leading to a relative enrichment of mutant cells. As mentioned, this fitness was not maintained as the relative numbers of mutants was increased. We speculate that this may be in part due to a lack of WT for the mutant to exploit. With smaller relative numbers of WT cells present to be exploited by a larger relative number of mutant cells, it will be increasingly harder for the latter to gain any beneficial advantages from the WT, as has been suggested previously (Diggle et al., 2007). Furthermore, having far more degenerated, acid producing cells such as *spo0A* mutants (Supplementary Figure 2) would have a far more detrimental effect on the culture, due to previously reported acid toxicity (Maddox et al., 2000). With increasing mutant to WT ratios, the latter will no longer be able to offset the continued production of acids by degenerated cells, upon which the fitness advantage of the mutants is reduced and eventually lost.

As one might predict, we were able to prevent the degeneration process through adjusting the transfer times or applying a bottleneck. By transferring the culture past the point where we observed that degenerate mutant cells had begun to die (after 24 h, Supplementary Figure 5), it may be assumed that any remaining WT cells that had not undergone a transition to spores, could grow once again in fresh medium. Alternatively, by applying a heat shock, any degenerated cell unable to form viable spores would be killed, allowing exclusive propagation of spore-forming (and solvent producing) members of the population. Both these approaches allowed for maintenance of WT characteristics and variations of these techniques have been used for industrial fermentations in the past. For example, during the scale-up process to reach volumes of industrial needs, several starter cultures were used which increase in volume (Nimcevic and Gapes, 2000). Heat shocking of these cultures was employed to create healthy spore-seed cultures when ABE fermentation was still utilized for industrial scale solvent production. Several heat shock cycles were employed until a final culture containing high levels of healthy spores was created and used as the initial seed culture for scale up (Jones, 2000). It should be noted however that this was only employed for the creation of the starter seed culture, and repeated heat shocks during scale was not utilized. Furthermore, extensive time between subcultures (bimonthly) has also been employed to maintain *C. beijerinckii* stock cultures that were always spore derived (Stephens et al., 1985). Finally, by investigating the reasons for the *spo0A* mutants’ demise past the 24-h time point, in contrast to non-sporulating WT cells which remained viable (Supplementary Figure 5), new ways of eliminating non-solvent-producing cells may be found.

This study has established the genetic and evolutionary basis of strain degeneration in the solvent producing species *C. beijerinckii* NCIMB 8052 by establishing the role of the master regulator gene *spo0A*. Genetic and evolutionary experimentation revealed that both partial and complete loss of Spo0A activity results in a fitness benefit. Mutants are therefore able to propagate throughout the population and outcompete the wild type. These mutants usually lack the ability to produce solvents and spores effectively making them undesirable in a commercial fermentation. Degeneration still poses a problem for industry but with a greater understanding of the genes involved, it is hoped that prevention can be achieved.

## Materials and methods

### Bacterial strains, growth media, and culture conditions

Bacterial strains utilized in this study are listed in Table 1*. Clostridium beijerinckii* NCIMB 8052 and generated mutants were grown at 37°C in an anaerobic cabinet (MG1000 Anaerobic Work Station, Don Whitley Scientific) containing an atmosphere of 80% nitrogen, 10% hydrogen and 10% carbon dioxide. The organism was routinely cultured in supplemented clostridial basal medium (CBMS), unless stated otherwise. CBMS was based on CBM as previously described (O’Brien and Morris, 1971) but contained glucose (60 g/L unless otherwise stated) and calcium carbonate (5 g/L) as a buffering agent. For agar plates, 15 g/L technical agar 1 (Oxoid, United Kingdom) was added. *Escherichia coli* TOP10 was grown in lysogeny broth (LB) at 37°C. Antibiotics were used at the following concentrations: erythromycin, 500 μg/ml for *E. coli* and 10 μg/ml for *C. beijerinckii*; spectinomycin 250 μg/ml for *E. coli* and 750 μg/ml for *C. beijerinckii*. *Clostridium beijerinckii* wild type was stored as a spore stock and generated mutants as 10% (v/v) glycerol stocks frozen at −80°C.

**Table 1:** Bacterial strains utilized in this study.

Strain	Properties	Source
*Escherichia coli* TOP10	Cloning *E.coli* strain	Invitrogen
*Clostridium beijerinckii* NCIMB 8052	Wild type *C. beijerinckii* NCIMB 8052	NCIMB
*C. beijerinckii* NCIMB 8052 parental wild type 1	*C. beijerinckii* 8052 parental wild type for the first set of degenerates	This Study
*C. beijerinckii* NCIMB 8052 parental wild type 2	*C. beijerinckii* 8052 parental wild type for the second set of degenerates	This Study
*C. beijerinckii* 8052 RD1	Isolated *C. beijerinckii* 8052 degenerate RD1 (first set)	This Study
*C. beijerinckii* 8052 RD2	Isolated *C. beijerinckii* 8052 degenerate RD2 (first set)	This Study
*C. beijerinckii* 8052 RD3	Isolated *C. beijerinckii* 8052 degenerate RD3 (first set)	This Study
*C. beijerinckii* 8052 RD4	Isolated *C. beijerinckii* 8052 degenerate RD4 (first set)	This Study
*C. beijerinckii* 8052 RD5	Isolated *C. beijerinckii* 8052 degenerate RD5 (first set)	This Study
*C. beijerinckii* 8052 RD1-1	Isolated *C. beijerinckii* 8052 degenerate RD1-1 (second set)	This Study
*C. beijerinckii* 8052 RD1-2	Isolated *C. beijerinckii* 8052 degenerate RD1-2 (second set)	This Study
*C. beijerinckii* 8052 RD2-1	Isolated *C. beijerinckii* 8052 degenerate RD2-1 (second set)	This Study
*C. beijerinckii* 8052 RD2-2	Isolated *C. beijerinckii* 8052 degenerate RD2-2 (second set)	This Study
*C. beijerinckii* 8052 RD3-1	Isolated *C. beijerinckii* 8052 degenerate RD3-1 (second set)	This Study
*C. beijerinckii* 8052 RD3-2	Isolated *C. beijerinckii* 8052 degenerate RD3-2 (second set)	This Study
*C. beijerinckii* 8052 RD4-1	Isolated *C. beijerinckii* 8052 degenerate RD4-1 (second set)	This Study
*C. beijerinckii* 8052 RD4-2	Isolated *C. beijerinckii* 8052 degenerate RD4-2 (second set)	This Study
*C. beijerinckii* 8052 RD5-1	Isolated *C. beijerinckii* 8052 degenerate RD5-1 (second set)	This Study
*C. beijerinckii* 8052 RD5-2	Isolated *C. beijerinckii* 8052 degenerate RD5-2 (second set)	This Study
*C. beijerinckii* 8052 RD6-1	Isolated *C. beijerinckii* 8052 degenerate RD6-1 (second set)	This Study
*C. beijerinckii* 8052 RD6-2	Isolated *C. beijerinckii* 8052 degenerate RD6-2 (second set)	This Study
*C. beijerinckii* 8052 CIC1	Isolated *C. beijerinckii* 8052 degenerate CIC1 (first set)	This Study
*C. beijerinckii* 8052 CIC2	Isolated *C. beijerinckii* 8052 degenerate CIC2 (first set)	This Study
*C. beijerinckii* 8052 CIC3	Isolated *C. beijerinckii* 8052 degenerate CIC3 (first set)	This Study
*C. beijerinckii* 8052 CIC4	Isolated *C. beijerinckii* 8052 degenerate CIC4 (first set)	This Study
*C. beijerinckii* 8052 CIC5	Isolated *C. beijerinckii* 8052 degenerate CIC5 (first set)	This Study
*C. beijerinckii* 8052 CIC6	Isolated *C. beijerinckii* 8052 degenerate CIC6 (first set)	This Study
*C. beijerinckii* 8052 CIC1-1	Isolated *C. beijerinckii* 8052 degenerate CIC1-1 (second set)	This Study
*C. beijerinckii* 8052 CIC1-2	Isolated *C. beijerinckii* 8052 degenerate CIC1-2 (second set)	This Study
*C. beijerinckii* 8052 CIC2-1	Isolated *C. beijerinckii* 8052 degenerate CIC2-1 (second set)	This Study
*C. beijerinckii* 8052 CIC2-2	Isolated *C. beijerinckii* 8052 degenerate CIC2-2 (second set)	This Study
*C. beijerinckii* 8052 CIC3-1	Isolated *C. beijerinckii* 8052 degenerate CIC3-1 (second set)	This Study
*C. beijerinckii* 8052 CIC3-2	Isolated *C. beijerinckii* 8052 degenerate CIC3-2 (second set)	This Study
*C. beijerinckii* 8052 CIC4-1	Isolated *C. beijerinckii* 8052 degenerate CIC4-1 (second set)	This Study
*C. beijerinckii* 8052 CIC4-2	Isolated *C. beijerinckii* 8052 degenerate CIC4-2 (second set)	This Study
*C. beijerinckii* 8052 CIC5-1	Isolated *C. beijerinckii* 8052 degenerate CIC5-1 (second set)	This Study
*C. beijerinckii* 8052 CIC5-2	Isolated *C. beijerinckii* 8052 degenerate CIC5-2 (second set)	This Study
*C. beijerinckii* 8052 CIC6-1	Isolated *C. beijerinckii* 8052 degenerate CIC6-1 (second set)	This Study
*C. beijerinckii* 8052 CIC6-2	Isolated *C. beijerinckii* 8052 degenerate CIC6-2 (second set)	This Study
*C. beijerinckii* 8052 DCOG1	Isolated *C. beijerinckii* 8052 degenerate DCOG1 (first set)	This Study
*C. beijerinckii* 8052 DCOG2	Isolated *C. beijerinckii* 8052 degenerate DCOG2 (first set)	This Study
*C. beijerinckii* 8052 DCOG3	Isolated *C. beijerinckii* 8052 degenerate DCOG3 (first set)	This Study
*C. beijerinckii* 8052 DCOG4	Isolated *C. beijerinckii* 8052 degenerate DCOG4 (first set)	This Study
*C. beijerinckii* 8052 DCOG5	Isolated *C. beijerinckii* 8052 degenerate DCOG5 (first set)	This Study
*C. beijerinckii* 8052 DCOG1-1	Isolated *C. beijerinckii* 8052 degenerate DCOG1-1 (second set)	This Study
*C. beijerinckii* 8052 DCOG1-2	Isolated *C. beijerinckii* 8052 degenerate DCOG1-2 (second set)	This Study
*C. beijerinckii* 8052 DCOG2-1	Isolated *C. beijerinckii* 8052 degenerate DCOG2-1 (second set)	This Study
*C. beijerinckii* 8052 DCOG2-2	Isolated *C. beijerinckii* 8052 degenerate DCOG2-2 (second set)	This Study
*C. beijerinckii* 8052 DCOG3-1	Isolated *C. beijerinckii* 8052 degenerate DCOG3-1 (second set)	This Study
*C. beijerinckii* 8052 DCOG3-2	Isolated *C. beijerinckii* 8052 degenerate DCOG3-2 (second set)	This Study
*C. beijerinckii* 8052 DCOG4-1	Isolated *C. beijerinckii* 8052 degenerate DCOG4-1 (second set)	This Study
*C. beijerinckii* 8052 DCOG4-2	Isolated *C. beijerinckii* 8052 degenerate DCOG4-2 (second set)	This Study
*C. beijerinckii* 8052 DCOG5-1	Isolated *C. beijerinckii* 8052 degenerate DCOG5-1 (second set)	This Study
*C. beijerinckii* 8052 DCOG5-2	Isolated *C. beijerinckii* 8052 degenerate DCOG5-2 (second set)	This Study
*C. beijerinckii* 8052 DCOG6-1	Isolated *C. beijerinckii* 8052 degenerate DCOG6-1 (second set)	This Study
*C. beijerinckii* 8052 DCOG6-2	Isolated *C. beijerinckii* 8052 degenerate DCOG6-2 (second set)	This Study
*C. beijerinckii* 8052 FW1	Isolated *C. beijerinckii* 8052 degenerate FW1 (first set)	This Study
*C. beijerinckii* 8052 FW2	Isolated *C. beijerinckii* 8052 degenerate FW2 (first set)	This Study
*C. beijerinckii* 8052 FW3	Isolated *C. beijerinckii* 8052 degenerate FW3 (first set)	This Study
*C. beijerinckii* 8052 FW4	Isolated *C. beijerinckii* 8052 degenerate FW4 (first set)	This Study
*C. beijerinckii* 8052 FW5	Isolated *C. beijerinckii* 8052 degenerate FW5 (first set)	This Study
*C. beijerinckii* 8052 FW6	Isolated *C. beijerinckii* 8052 degenerate FW6 (first set)	This Study
*C. beijerinckii* 8052 FW7	Isolated *C. beijerinckii* 8052 degenerate FW7 (first set)	This Study
*C. beijerinckii* 8052 FW1-1	Isolated *C. beijerinckii* 8052 degenerate FW1-1 (second set)	This Study
*C. beijerinckii* 8052 FW1-2	Isolated *C. beijerinckii* 8052 degenerate FW1-2 (second set)	This Study
*C. beijerinckii* 8052 FW2-1	Isolated *C. beijerinckii* 8052 degenerate FW2-1 (second set)	This Study
*C. beijerinckii* 8052 FW2-2	Isolated *C. beijerinckii* 8052 degenerate FW2-2 (second set)	This Study
*C. beijerinckii* 8052 FW3-1	Isolated *C. beijerinckii* 8052 degenerate FW3-1 (second set)	This Study
*C. beijerinckii* 8052 FW3-2	Isolated *C. beijerinckii* 8052 degenerate FW3-2 (second set)	This Study
*C. beijerinckii* 8052 FW4-1	Isolated *C. beijerinckii* 8052 degenerate FW4-1 (second set)	This Study
*C. beijerinckii* 8052 FW4-2	Isolated *C. beijerinckii* 8052 degenerate FW4-2 (second set)	This Study
*C. beijerinckii* 8052 FW5-1	Isolated *C. beijerinckii* 8052 degenerate FW5-1 (second set)	This Study
*C. beijerinckii* 8052 FW5-2	Isolated *C. beijerinckii* 8052 degenerate FW5-2 (second set)	This Study
*C. beijerinckii* 8052 FW6-1	Isolated *C. beijerinckii* 8052 degenerate FW6-1 (second set)	This Study
*C. beijerinckii* 8052 FW6-2	Isolated *C. beijerinckii* 8052 degenerate FW6-2 (second set)	This Study
*C. beijerinckii* 8052? pyrE	*C. beijerinckii* 8052 mutant with truncated pyrE gene	This Study
*C. beijerinckii* 8052? pyrE spo0A::CTermB	*C. beijerinckii* 8052 spo0A ClosTron mutant with truncated pyrE gene	This Study
*C. beijerinckii* 8052? pyrE Cbei_0017::CTermB	*C. beijerinckii* 8052 C.bei_0017 ClosTron mutant with truncated pyrE gene	This Study
*C. beijerinckii* 8052? pyrE Cbei_3078::CTermB	*C. beijerinckii* 8052 C.bei_3078 ClosTron mutant with truncated pyrE gene	This Study
*C. beijerinckii* 8052 spo0A::CTermB	*C. beijerinckii* 8052 spo0A ClosTron mutant with repaired pyrE gene	This Study
*C. beijerinckii* 8052 Cbei_0017::CTermB	*C. beijerinckii* 8052 C.bei_0017 ClosTron mutant with repaired pyrE gene	This Study
*C. beijerinckii* 8052 Cbei_3078::CTermB	*C. beijerinckii* 8052 C.bei_3078 ClosTron mutant with repaired pyrE gene	This Study
*C. beijerinckii* 8052 spo0A::CTermB_comp	*C. beijerinckii* 8052 spo0A ClosTron mutant with complemented gene at the pyrE locus	This Study
*C. beijerinckii* 8052 Cbei_0017::CTermB_comp	*C. beijerinckii* 8052 C.bei_0017 ClosTron mutant with complemented gene at the pyrE locus	This Study
*C. beijerinckii* 8052 Cbei_3078::CTermB_comp	*C. beijerinckii* 8052 C.bei_3078 ClosTron mutant with complemented gene at the pyrE locus	This Study

### Plasmids, primers, and DNA techniques

Plasmids and primers used in this study are listed in Tables 2, 3, respectively. Primers were synthesized by Sigma-Aldrich, United States. PCR amplifications were carried out using high fidelity Phusion polymerase (New England Biolabs, United States) or DreamTaq DNA polymerase (ThermoFischer Scientific, United States). Plasmid isolation carried out using Monarch Plasmid Miniprep Kit (New England Biolabs, United States) and genomic DNA preparations were carried out using the GenElute Bacterial Genomic DNA kit (Sigma-Aldrich, United States) following the manufactures instructions. Restriction enzymes were supplied by New England Biolabs and were used according to the manufacturers’ instructions. Electroporation of *C. beijerinckii* was performed as described previously (Oultram et al., 1988).

**Table 2:** Plasmids utilized in this study.

Plasmid	Properties	Source
pMTL83251	Clostridium modular plasmid containing a pCB102 Gram-positive replicon, a ermB resistance marker, ColE1 + TraJ Gram-negative replicon and a lacZ multiple cloning site	Heap et al. (2010)
pMTL-JRH1	pyrE truncation vector containing a pCB102 Gram-positive replicon, a ermB resistance marker, ColE1 + TraJ Gram-negative replicon and a lacZ multiple cloning site	This study
pMTL84351	Clostridium modular plasmid containing a pCD6 Gram-positive replicon, a aad9 resistance marker, a ColE1 + TraJ Gram-negative replicon and a lacZ multiple cloning site	Heap et al. (2010)
pMTL-JRH4	pyrE repair vector containing a pCD6 Gram-positive replicon, a aad9 resistance marker, a ColE1 + TraJ Gram-negative replicon and a lacZ multiple cloning site	This study
pMTL-JRH4_Cbei_0017	pMTL-JRH4 containing the Cbei_0017 gene and approximately 200 bp upstream for the promoter region	This study
pMTL-JRH4_spo0A	pMTL-JRH4 containing the spo0A gene and approximately 200 bp upstream for the promoter region	This study
pMTL-JRH4_Cbei_3078	pMTL-JRH4 containing the Cbei_3078 gene and approximately 200 bp upstream for the promoter region	This study
pMTL007S-E2::cbei_0017–694|695a	ClosTron plasmid targetting the Cbei_0017 gene	ATUM
pMTL007S-E2::cbei_3078–969|970 s	ClosTron plasmid targetting the Cbei_3078 gene	ATUM
pMTL007S-E2::cbei_3077–138|139a	ClosTron plasmid targetting the Cbei_3077 gene	ATUM
pMTL007S-E2::cbei_4884–159|160 s	ClosTron plasmid targetting the Cbei_4884 gene	ATUM
pMTL007S-E2::cbei_4885–108|109 s	ClosTron plasmid targetting the Cbei_4885 gene	ATUM
pMTL007S-E2::Cbe-spo0A-407a	ClosTron plasmid targetting the spo0A gene	Heap et al. (2010)

**Table 3:** Primers utilized in this study.

Oligonucleotides	Use in this study	Sequence
pyrE KO LHA_F	Generation of the 300 bp LHA for pyrE KO using HiFi assembly	CAGGAAACAGCTATGACCGCATGAAGGCATATAAGAAAGAATTTATC
pyrE KO LHA_R	Generation of the 300 bp LHA for pyrE KO using HiFi assembly	CTTAATACTTTTATCTATTTGAACAATATCTTACATC
pyrE KO RHA_F	Generation of the 1,200 bp RHA for pyrE KO using HiFi assembly	CAAATAGATAAAAGTATTAAGAACAACTCAACGTG
pyrE KO RHA_R	Generation of the 1,200 bp RHA for pyrE KO using HiFi assembly	CATGGTCATATGGATACAGCGGAATGTTAAGATTTAATACCAC
pyrE KO confirmation_F	Confirmation that the pyrE of *C. beijerinckii* NCIMB 8052 had been truncated	CAAAGTAGATATTGGAGGAATT
pyrE KO confirmation_R	Confirmation that the pyrE of C. beijerinckii NCIMB 8052 had been truncated	AACACTTGCAGTCTTATGAAC
pyrE repair LHA_F	Generation of the intact pyrE gene for repair. Contains an application module for future complementation	GGTTCCTGCAGGATGAAGGCATATAAGAAAGAAT
pyrE repair LHA_R	Generation of the intact pyrE gene for repair. Contains an application module for future complementation	CGCGGCGGCCGCTTATTTTGCACCATATTGTTT
pyrE repair RHA_F	Generation of the intact pyrE gene for repair. Contains an application module for future complementation	GGTTGCTAGCAAGTATTAAGAACAACTCAACG
pyrE repair RHA_R	Generation of the intact pyrE gene for repair. Contains an application module for future complementation	CCCCGGCGCGCCGGAATGTTAAGATTTAATACCAC
spo0A gene and promoter_F	spo0A gene and its native promoter for complementation	CGCGCATATGAACAAATGATGGGTAGCGTATT
spo0A gene and promoter_R	spo0A gene and its native promoter for complementation	GGCCGGATCGCAATTAGCTAACTTTATTTTTAAGTCTTAA
Cbei_0017 gene and promoter_F	Cbei_0017 gene and its native promoter for complementation	GGCCGCGGCCGCTAATATAAGCATATAT
Cbei_0017 gene and promoter_R	Cbei_0017 gene and its native promoter for complementation	CGCGCATATGCTTTTACAAATATATATCA
Cbei_3077 gene and promoter_F	Cbei_3077 gene and its native promoter for complementation	AATTGCGGCCGAGGCTTATAAGGCAGCACTAATA
Cbei_3077 gene and promoter_R	Cbei_3077 gene and its native promoter for complementation	GGCCGGATCCAACTTACATTAATTCAAAAAATATATCC
Cbei_3078 gene and promoter_F	Cbei_3078 gene and its native promoter for complementation	AATTGCGGCCGCGAATGAAAGAGTGTGTTATAAAAATAAT
Cbei_3078 gene and promoter_R	Cbei_3078 gene and its native promoter for complementation	GCGCGGATCCCCTCTATGTTCTTAAGTTGAATTCTATA
Cbei_4884 gene and promoter_F	Cbei_4884 gene and its native promoter for complementation	AATTGCGGCCGCAAAAATTCCTCCTAATTCCAT
Cbei_4884 gene and promoter_R	Cbei_4884 gene and its native promoter for complementation	GGCCGGATCCAATTTATTCTTCTTCATACTCTTTAACT
Cbei_4885 gene and promoter_F	Cbei_4885 gene and its native promoter for complementation	AATTGCGGCCGCTATCATGCACCTATTCTTTCTCAAT
Cbei_4885 gene and promoter_R	Cbei_4885 gene and its native promoter for complementation	GGCCGGATCCATACTATCTGTTTGTTTTAATTTCGTCT
spo0A check_F	Confirmation primers of clostron insertion and for SNVs detection	AGAATGGACAAAAGGAGAGA
spo0A check_R	Confirmation primers of clostron insertion and for SNVs detection	CCTTCAACGTAGCTTTTCAT
Cbei_0017 check_F	Confirmation primers of clostron insertion and for SNVs detection	CTAAATTTGTAATATTCAATTATTAAAC
Cbei_0017 check_R	Confirmation primers of clostron insertion and for SNVs detection	ATATTCAATTTTCACAACAAGTT
Cbei_3078 check_F	Confirmation primers of clostron insertion and for SNVs detection	ATTGTATTTTTCCCCATTC
Cbei_3078 check_R	Confirmation primers of clostron insertion and for SNVs detection	ATAATATAAGCGAGGCCTT
Cbei_4884 check_F	Confirmation primers of clostron insertion and for SNVs detection	CCTAGCACTTCCAGAAAAC
Cbei_4884 check_R	Confirmation primers of clostron insertion and for SNVs detection	TAAAGTAATATAAGGCATATGCC
Cbei_4885 check_F	Confirmation primers of clostron insertion and for SNVs detection	CTCTAACCATTGGTACATCTAG
Cbei_48845 check_R	Confirmation primers of clostron insertion and for SNVs detection	ACTTGAAGAGATAAAGGAAGAG

### Construction of mutants using ClosTron technology

*Clostridium beijerinckii* NCIMB 8052 ClosTron mutagenesis was carried out as described by Heap et al. (Hayashida and Yoshino, 1989; West et al., 2006). ClosTron carrying plasmids were designed using the design tool available on http://www.ClosTron.com/ClosTron2.php and purchased from ATUM (formerly DNA 2.0). Numbers in the respective plasmid names (Table 2) indicate the retargeting site used within the disrupted gene. Genomic DNA from putative mutants was screened by PCR using primers that flanked the gene (see Supplementary Table 1) to establish whether the ClosTron-derived group II intron had inserted at the desired site. The generated PCR fragments were Sanger sequenced to obtain definite proof that intron insertion had occurred at the desired position.

### Sanger, whole and ultra-deep sequencing

Sanger sequencing was carried out by Source BioScience (Nottingham, United Kindom). Isolated genomic DNA was whole genome sequenced by MicrobesNG (Birmingham, United Kindom). Genome sequences were aligned and compared using CLC genomics workbench v.10 (Qiagen, Germany). Ultra-deep sequencing was carried out by Deepseq (Nottingham, UK.) and sequencing libraries were prepared using the KAPA HyperPlus PCR-free Kit (Roche; KK8513) and the KAPA Dual-Indexed Adapter Kit, Illumina Platforms (Roche; KK8722). Genomic DNA was quantified using the Qubit Fluorometer and the Qubit dsDNA BR kit (ThermoFisher; Q32853) and 500 ng of each sample were used for library preparation. Genomic DNA was fragmented for 10 min and the PCR-free workflow was followed, according to the KAPA HyperPlus Kit protocol (KAPA Biosystems; KR1145—v3.16). The final double-sided SPRI-based size selection was performed using 0.7/0.9 ratios of AMPure XP beads (Beckman Coulter; A63882). Libraries were quantified using the Qubit Fluorometer and the Qubit dsDNA HS Assay Kit (ThermoFisher: Q32854) and fragment length distributions were assessed using the Agilent 2,100 Bioanalyzer and the Agilent High Sensitivity DNA Kit (Agilent; 5067-4626). Libraries were pooled in equimolar amounts and final library quantification was performed using the KAPA Library Quantification Kit for Illumina (Roche; KK4824). The library pool was sequenced on the Illlumina NextSeq 500 using a NextSeq 500 High Output 300 cycle kit v2.5 (Illumina; 20024908), to generate 150-bp paired-end reads.

Variant calling: Reads were trimmed using Cutadapt (Martin, 2011) v2.10 with parameters *-A* “*AGATCGGAAGAGCACACGTC*” *-a* “*AGATCGGAAGAGCACACGTC*” *--trim-n --nextseq-trim = 20 -m 30*. Trimmed reads were aligned with BWA-MEM (Li and Durbin, 2009) v0.7.17 to *C. beijerinckii* reference (NCIMB 8052 ASM1696v1). Variant calling was performed using FreeBayes (Garrison and Marth, 2012) v1.3.2 and LoFreq (Wilm et al., 2012) v2.1.5. We performed Freebayes calling using parameters *-J --no-population-priors -p 50 -F 0.005 -C 2 --min-base-quality 20 --use-best-n-alleles 4 --use-duplicate-reads*. Lofreq calling was performed with default parameters. Multiallelic variant calls were split using bcftools (Danecek et al., 2021) v1.10.2 using parameters *-m -any*.

Joint-Called variant Filtering: SNPs called by FreeBayes with *QUAL ≥ 100* and LoFreq with *FILTER = PASS* were kept. For each joint-called variant, we generated a pileup in first-forward and first-reverse orientation using RustyNuc v0.3.0 (Debebe, 2021) using parameters *--min-reads 4 -q 20*. Only calls with AF above 0.04 in both first-forward and first-reverse orientation were kept (Supplementary Table 4A).

Heatmap filtering: Heatmap was created using ComplexHeatmap v2.12.0 (Gu et al., 2016) and we excluded variants that (i) did not occur in at least three subcultures with *AF > 0.08* in a single replicate, (ii) located in rRNA regions and (iii) already occurred early on in the starter culture (subculture 0 with *AF > 0.5*). In addition, we removed variant call at position 1,957,725 since it displayed similar dynamics across replicates, occurred in majority of subcultures and displayed correlation with sequencing depth across replicates (Supplementary Table 4C).

Genome Annotations: operon and transcription factor binding site locations were obtained from *OperonDB* (Pertea et al., 2009), *CollecTF* (Kiliç et al., 2014) and *PRODORIC* (Dudek and Jahn, 2022).

All raw data used for the ultra-deep sequencing has been deposited at the European Nucleotide Archive (ENA) with accession PRJEB51942 and can be accessed *via* https://lowfreqvar.github.io.

### Subculturing protocols

A full schematic of the subculturing protocol can be found in Supplementary Figure 1, but generally was performed as follows; 50 μl of N-WT spore suspension was heated at 80°C for 10 min in a heating block, serially diluted down to 10^−3^ in sterile PBS, plated on CBMS agar and left to germinate overnight at 37°C in the anaerobic work station (MG1000 Anaerobic Work Station, Don Whitley Scientific). Single colonies were then inoculated into 10 ml CBMS liquid medium and left for 24 h. 1 ml of culture was inoculated into fresh 9 ml CBMS liquid medium in a 1:10 dilution every 24 h for however many subcultures each experiment required.

For isolation of potential degenerates, a single germinated colony was mixed with 1 ml CBMS liquid medium and 100 μl of the suspension was distributed to 6 parallel tubes containing 10 ml CBMS liquid medium. The remaining liquid was left for 24 h and served as the parental wild type genomic profile. Parallel cultures were subcultured 5 times as previously described. Afterwards, 100 μl of culture was serially diluted 1:10 down to 10^−5^ with anaerobic PBS, plated on CBMS agar and incubated for 2 days to give single colonies. Colony variants were selected based on their colony morphology and inoculated into 10 ml CBMS liquid medium, left overnight and afterwards a 1 ml sample was taken and made into a 10% (v/v) glycerol stock for future use. Genomic DNA was then isolated for subsequent analysis.

To study culture dynamics with respect to culture productivity (organic solvents, acids and spores) and colony morphologies, the standard subculturing protocol as previously described was employed. 1 ml fermentation product samples were taken at the point of inoculation and 2 days after inoculation at each successive subculture. These were centrifuged at full speed for 1 min and the supernatant removed and frozen at −20°C for future analysis. 1 ml spore samples were taken after 5 days of growth and stored at 4°C until used in a spore assay. To observe colony morphologies at each subculture, after 1 day of growth (post inoculation), 200 μl samples were taken, serially diluted down to 10–5 and plated onto CBMS agar. Colonies were left to grow for 2 days and the CFU of each colony type was enumerated. Transfer times were changed from 24 to 72 h for one experiment. The 1 ml transfer culture was also heat shocked at 80°C before subculturing for one experiment.

### Fitness experiments

Wild type spores were heat shocked as described for the above subculturing protocol and plated on CBMS agar and desired degenerated variants were streaked onto CBMS agar from a frozen stock. Each were left to grow for 24 h. Colonies were inoculated into 10 ml CBM 6% glucose broth and left to grow for 24 h. 24 h cultures of WT and the desired degenerate were added in varying volumetric ratios to make a total of 1 ml which was used to inoculate to 9 ml CBM 6% glucose liquid so that the final volume was 10 ml. For example, 100 μl degenerate variant +900 μl wild type added to 9 ml CBM-S broth 6% glucose. This would give a theoretical starting ratio of 1:10 (degenerate:WT) in the 1 ml inoculum, with exact ratios determined experimentally through enumeration of colony morphotypes (CFU/ml) following spread-plating. CFU/ml were enumerated at 0, 12, 24, 48, and 72 h using serial dilutions of the culture. Fitness of individuals was calculated using *w* = [*x_2_*(1 *– x_1_*)]/[*x_1_*(1 *– x_2_*)] from Diggle et al.^45^ where *x_1_* is the initial proportion of mutant in the population and *x_2_* is the final proportion at a given time point.

### Analytical techniques

Gas chromatography (GC) was used for acetone, ethanol, butanol, acetate and butyrate concentration analysis in which 1 ml fermentation samples were taken, centrifuged at 16,000 ×g for 1 min with the supernatant removed and stored at −20°C until needed. Extraction of fermentation products and their gas chromatographic analysis was carried out as described previously (Willson et al., 2016). Optical density at 600 nm was used as a measure of cell growth and determined using a Thermo Scientific Biomate 3 spectrophotometer with sterile liquid medium as a blank. If samples had OD600 above 0.8, samples were diluted 1:10 and re-measured.

## Data availability statement

The data presented in the study are deposited in the European Nucleotide Archive (ENA), accession PRJEB51942 and at https://lowfreqvar.github.io.

## Author contributions

JH, BD, SD, and KW designed the research. JH, SD, and KW analyzed the microbiological data. BD: genetic data. JH and BD performed the research. All authors contributed to the article and approved the submitted version.

## Funding

This work was supported by the Biotechnology and Biological Sciences Research Council (BBSRC; Grant numbers BB/L013940/1 and BB/J014508/1); and the Engineering and Physical Sciences Research Council (EPSRC; Grant number BB/L013940/1). We thank The University of Nottingham for supporting the PhD studentship of JH.

## Conflict of interest

The authors declare that the research was conducted in the absence of any commercial or financial relationships that could be construed as a potential conflict of interest.

## Publisher’s note

All claims expressed in this article are solely those of the authors and do not necessarily represent those of their affiliated organizations, or those of the publisher, the editors and the reviewers. Any product that may be evaluated in this article, or claim that may be made by its manufacturer, is not guaranteed or endorsed by the publisher.
