# Supplementary material for: Clostridium beijerinckii strain degeneration is driven by the loss of Spo0A activity
Source: Front Microbiol. 2023 Jan 10;13:1075609. doi: 10.3389/fmicb.2022.1075609 (PMC9871927; doi:10.3389/fmicb.2022.1075609)
Supplement: Supplementary file 1 [file Data_Sheet_1.DOCX]

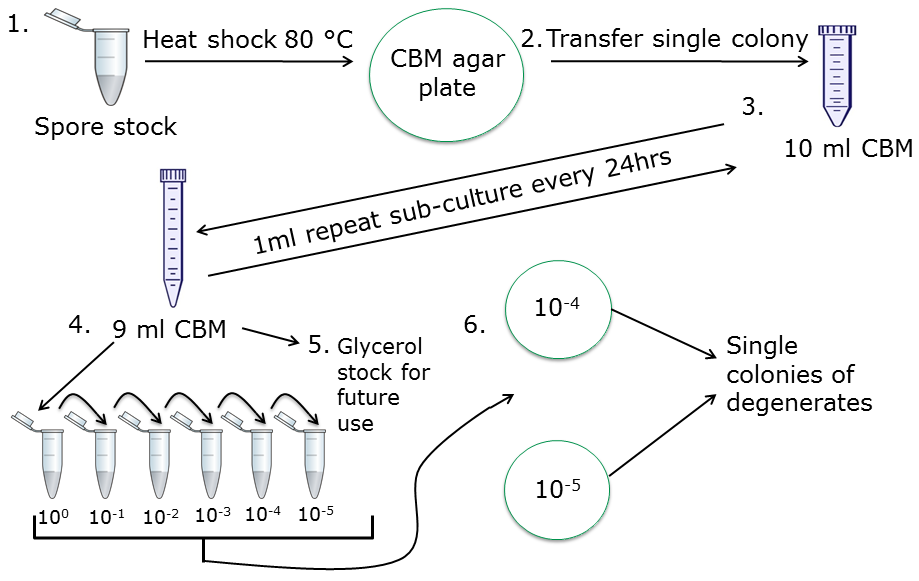


**SI figure 1. Subculturing schematic.**

A schematic of the subculturing process starting with heat shocking a WT spore stock, plating for single colonies, repeated subculturing and then analysis of colonies.

**B**

**C**

**A**

**D**

**SI figure 2. Fermentation profiles for isolated colonies.**

Solvent and acids produced by (A) round and dark (RD) colonies, (B) dark centre with outgrowths (DCOG) colonies, (C) caved in centre (CIC) colonies and (D) flat and white (FW) colonies. Values are the means (± SEM) of 6 replicates.

**B**

**C**

**A**

**D**

**SI figure 3. Sporulation profiles for isolated colonies.**

Spores produced by (A) round and dark (RD) colonies, (B) dark centre with outgrowths (DCOG) colonies, (C) caved in centre (CIC) colonies and (D) flat and white (FW) colonies. Values are the means (± SEM) of 6 replicates.


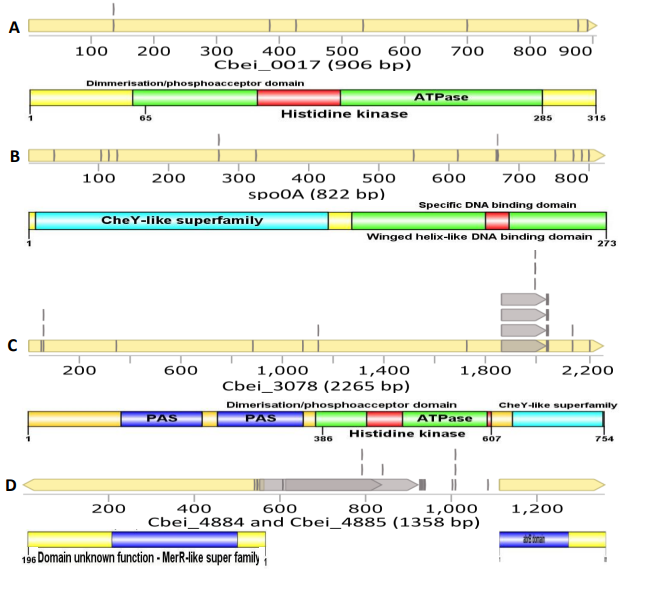


**SI figure 4. Location of mutations within the genes and the protein.**

(A) Cbei_0017. (B) *spo0A*. (C) Cbeki_3078. (D) Cbei_4884 and Cbei_4885).

**SI figure 5. CFU/mL of WT and FW7 when mixed in a 100:1 WT:FW ratio.**

CFU/mL of the WT and FW7 when mixed alongside % of FW7 in the population. Values are the means (± SEM) of 3 replicates.
